# Multimodal imaging observation of torpedo maculopathy

**DOI:** 10.3205/oc000265

**Published:** 2026-01-20

**Authors:** Hongtao Duan, Meng Dong, Shiyong Xie, Tongmei Zhang, Bin Wu, Yuezhong Zheng

**Affiliations:** 1 Tianjin Eye Hospital, Tianjin, China

## Abstract

An 18-year-old male was discovered with an oval, 1PD-sized, well-defined lesion on the inferonasal fovea of the right eye. Multimodal imaging was carried out. The lesion was diagnosed as torpedo maculopathy (TM). Most TM patients have no visual symptoms, are often detected during routine examination, and are generally stable.

## Introduction

Torpedo maculopathy (TM) is a rare, congenital condition affecting the retinal pigment epithelium (RPE) layer. The terminology was first coined by Daily [1]. Roseman and Gass [2] earlier described a similar lesion referred to as “RPE hypochromic nevus”. Typically, TM is on the macula’s temporal side, tip towards the fovea.

## Case description

**Figure 1:**
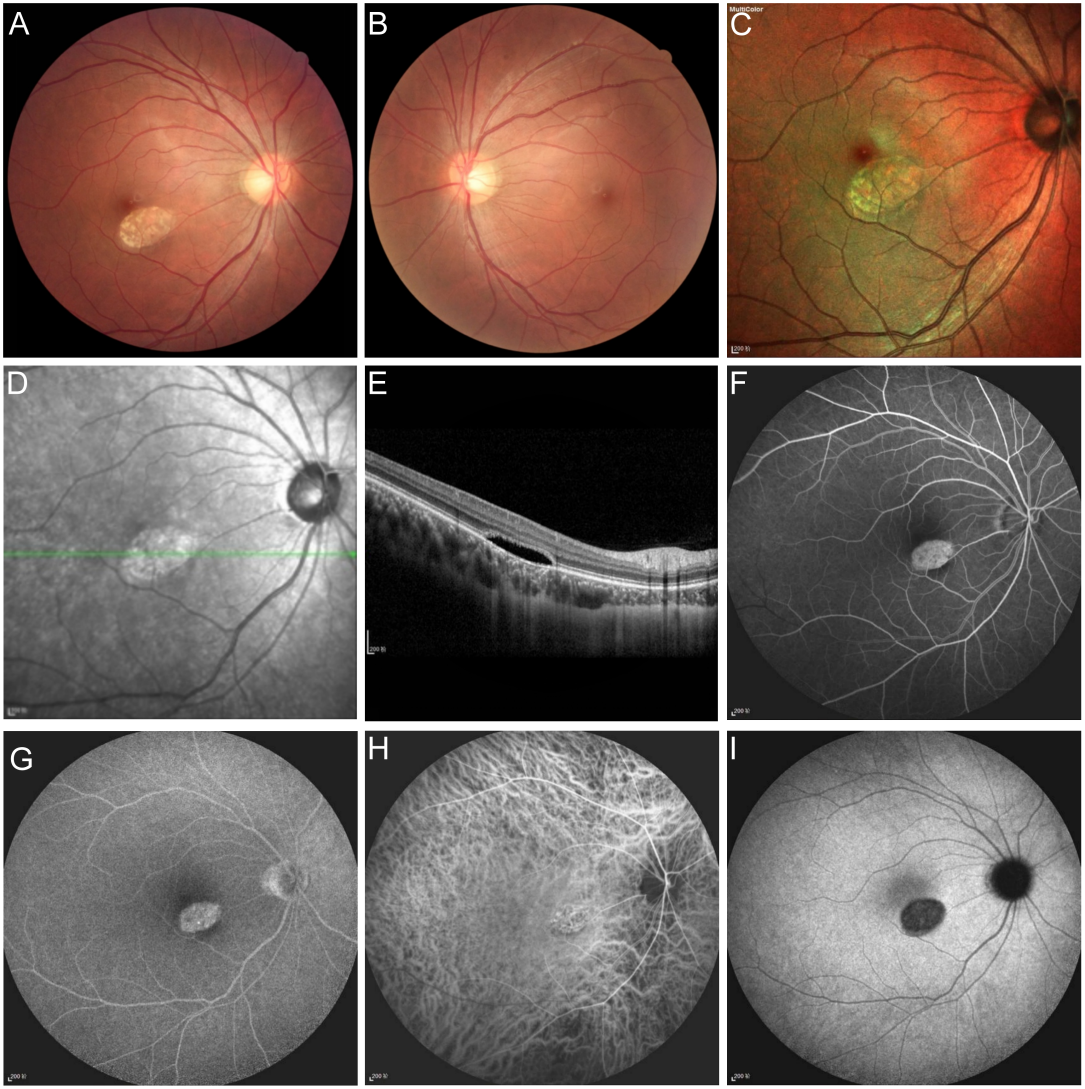
Multimodal imaging of the fundus in a patient with TM

An 18-year-old male was found with a fundus lesion due to physical examination. The visual acuity of both eyes was 1.0. He had no significant medical history, was born full-term, and had no family history of eye disorders. Both intraocular pressure and anterior segments were normal. In the right eye, an oval, 1PD-sized lesion was seen on the inferonasal fovea. The lesion had a slight acute angle along its long axis and contained irregular yellow-white dots. The retinal blood vessels over the lesion appeared normal. The fundus of the left eye was normal (Figure 1A–D (Fig. 1)). Optical coherence tomography (OCT) revealed structural disturbances in the outer layer of the retina, characterized by cleavage and a slightly enhanced choroidal reflex beneath the lesion (Figure 1E (Fig. 1)). In the early stage of fluorescein angiography, an oval-shaped hyperfluorescent lesion was observed, which remained hyperfluorescent in the late stage without leakage (Figure 1F, G (Fig. 1)). Indocyanine green fundus angiography demonstrated translucent choroidal vessels in the early stage, followed by hypofluorescent changes in the late stage (Figure 1H, I (Fig. 1)). Based on these findings, the diagnosis was confirmed as torpedo maculopathy (TM). He was advised to undergo regular follow-up examinations.

## Discussion

The exact etiology of TM remains unclear. Shields et al. [3] hypothesized that arose from improper development of the RPE in the fetus, potentially influenced by the location and size of lesion. The congenital nature of the lesion, as concluded by Pian et al. [4], resulted from incomplete differentiation of the arcuate tract along the horizontal median suture. This could lead to unusual shapes, such as torpedo-shaped lesion. Additionally, it had been proposed that disorders of the short ciliary arteries and veins, occurring before and after birth, might cause choroidal vascular dysplasia. It could damage RPE and contribute to the torpedo-like change [5]. Recently, OCT angiography have identified the loss of the choroidal capillary layer in TM, providing support for this hypothesis. Furthermore, a congenital choroidal valgus hypothesis was proposed to explain that.

Wong et al. [6] classified TM into two types based on characteristics identified through OCT. Type I TM presents with no outer retinal space and only minor disturbances in the outer retina, as well as possible thinning of the outer nuclear layer. Type II TM, on the other hand, shows a structural disturbance in the outer retina characterized by a cleavage. In both types, the inner layer of the retina remains normal, while the reflected signal through the choroid is increased. It is important to note that this classification does not account for TM with structural alterations in the inner layer of the retina. Consequently, Type III TM, was characterized by a distinctive fundoscopic appearance and no subretinal space on OCT, and may be associated with retinal choroidal depression, retinal thinning [7], [8], [9].

Most patients with TM do not exhibit visual symptoms. They generally remain stable during follow-up [10]. TM can also occur alongside other conditions, such as retinoblastoma [11], and congenital ocular toxoplasmosis [12]. Patients without complications typically do not require treatment, and regular follow-up is sufficient for monitoring their condition. However, chorioretinal atrophy in the deep layer may result in complications like choroidal neovascularization and decreased visual acuity [13], [14].

## Conclusion

In conclusion, torpedo maculopathy (TM) is a rare eye condition that typically does not significantly affect visual acuity. Currently, our understanding of its etiology remains limited due to the small number of reported cases. With the continuous advancement of ophthalmic imaging technology, we anticipate gaining deeper insights into the pathological mechanisms, clinical characteristics, and natural course of this condition in the future.

## Notes

### Funding

This work was supported by Tianjin Key Medical Discipline Construction (TJYXZDXK-3-004A-3).

### Competing interests

The authors declare that they have no competing interests.
